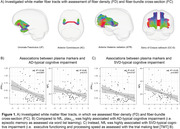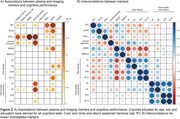# Understanding cognitive deficits in memory clinic patients with mixed disease by combining plasma markers and advanced diffusion MRI

**DOI:** 10.1002/alz.093710

**Published:** 2025-01-09

**Authors:** Anna Dewenter, Amir Dehsarvi, Anna Steward, Davina Biel, Julia Pescoller, Zeyu Zhu, Fabian Wagner, Mattes Gross, Brigitte Nuscher, Sebastian Niclas Roemer, Katharina Buerger, Daniel Janowitz, Michael Ewers, Steffen Tiedt, Martin Dichgans, Matthias Brendel, Nicolai Franzmeier

**Affiliations:** ^1^ Institute for Stroke and Dementia Research (ISD), University Hospital, LMU, Munich, Bavaria Germany; ^2^ Institute for Stroke and Dementia Research, LMU, Munich, Munich Germany; ^3^ Institute for Stroke and Dementia Research (ISD), University Hospital, LMU, Munich Germany; ^4^ Institute for Stroke and Dementia Research (ISD), LMU University Hospital, Munich, Munich (Bavaria) Germany; ^5^ German Center for Neurodegenerative Disorders, Munich Germany; ^6^ Department of Neurology, University Hospital, LMU, Munich, Bavaria Germany; ^7^ German Center for Neurodegenerative Diseases (DZNE), Munich, Bavaria Germany; ^8^ Institute for Stroke and Dementia Research (ISD), University Hospital, LMU,, Munich Germany; ^9^ German Center for Neurodegenerative Diseases (DZNE), Munich Germany; ^10^ LMU University Hospital, Munich Germany; ^11^ University of Gothenburg, The Sahlgrenska Academy, Institute of Neuroscience and Physiology, Psychiatry and Neurochemistry, Gothenburg Sweden

## Abstract

**Background:**

Memory clinic patients typically present with Alzheimer’s disease (AD) and cerebral small vessel disease (SVD) to varying degrees. Therefore, it is crucial to determine the etiology of cognitive deficits for facilitating patient‐centered treatment in memory clinics. Plasma biomarkers (ptau217, Glial Fibrillary Acidic Protein [GFAP], Neurofilament light chain [NfL]) and fixel‐based advanced diffusion MRI markers (fiber density, fiber‐bundle cross‐section) show potential towards disentangling AD‐ and SVD‐related brain changes (Dewenter et al., Brain, 2023). However, their predictive power in understanding heterogeneous and SVD/AD‐specific cognitive deficits in memory clinic patients remains incomplete. We assessed i) how plasma‐based and fixel markers explain AD‐typical and SVD‐typical cognitive deficits and ii) their interrelation to uncover disease‐specific mechanisms.

**Method:**

We included n = 76 in‐house memory clinic patients with Simoa‐based plasma ptau217, GFAP and NfL assessments, comprehensive neuropsychological testing (CERAD‐plus battery) and 3T MRI. Global white matter hyperintensity (WMH) volume and average skeletonized mean diffusivity (MD) were included as well‐established SVD markers. AD severity was probed through plasma ptau217 and cortical thickness of the pre‐established AD signature ROI. Advanced diffusion MRI was used to assess fiber density and fiber bundle cross‐section of key white matter tracts (Fig. 1A).

**Result:**

Using linear regression, increased ptau217, reduced cortical thickness in AD signature ROI and fiber‐bundle cross‐section were highly associated with impaired episodic memory, i.e. a typical AD‐related symptom (Fig.1B, Fig.2A). In contrast, GFAP and NfL increases and reductions in MSMD and fiber density were associated with executive dysfunction, a typical sign of SVD‐related cognitive impairment (Fig.1B, Fig.2A). Additionally, fiber density was associated with GFAP and NfL levels, while fiber bundle‐cross section, a macroscopic marker of tract atrophy, was not associated with any of the plasma markers (Fig.2B).

**Conclusion:**

Ptau217 displayed high sensitivity and specificity for AD‐typical memory impairment, whereas GFAP and NfL were associated with SVD‐typical processing speed and executive impairment. Additionally, fiber density, a pre‐established imaging marker for SVD, was associated with GFAP and NfL. This highlights the effectiveness of these markers in distinguishing and characterizing SVD and AD in memory clinic patients and emphasizes the importance of considering concomitant SVD in patients with elevated GFAP and NfL levels.